# Muscle and fat composition in patients with newly diagnosed multiple myeloma

**DOI:** 10.1038/s41408-023-00934-3

**Published:** 2023-12-12

**Authors:** Nadine H. Abdallah, Hiroki Nagayama, Naoki Takahashi, Wilson Gonsalves, Amie Fonder, Angela Dispenzieri, David Dingli, Francis K. Buadi, Martha Q. Lacy, Miriam Hobbs, Morie A. Gertz, Moritz Binder, Prashant Kapoor, Rahma Warsame, Suzanne R. Hayman, Taxiarchis Kourelis, Yi L. Hwa, Yi Lin, Robert A. Kyle, S. Vincent Rajkumar, Stephen M. Broski, Shaji K. Kumar

**Affiliations:** 1 Division of Hematology, Mayo Clinic, Rochester, MN USA; 2 Division of Radiology, Mayo Clinic, Rochester, MN USA

**Keywords:** Cancer imaging, Haematological cancer

## Abstract

Measures of muscle and adipose tissue mass have been associated with outcomes in several malignancies, but studies in multiple myeloma (MM) are inconsistent. The aim of this study was to evaluate the association between muscle and fat areas and radiodensity, and overall survival (OS) in patients with newly diagnosed MM. We included 341 patients diagnosed with MM from 2010–2019 who had an ^18^F-fluorodeoxyglucose positron emission tomography/computed tomography at diagnosis. A cross-sectional image at the third lumbar vertebrae was segmented into muscle and fat components. Median follow up was 5.7 years. There was no association between sarcopenia and baseline disease characteristics or OS. Low muscle radiodensity was associated with higher disease stage, anemia, and renal failure. OS was 5.6 vs. 9.0 years in patients with muscle radiodensity in the lower vs. middle/upper tertiles, respectively (*P* = 0.02). High subcutaneous adipose tissue (SAT) radiodensity was associated with higher stage, anemia, thrombocytopenia, hypercalcemia, renal failure, and high LDH. OS was 5.4 years vs. not reached in patients with SAT radiodensity in the upper vs. middle/lower tertiles, respectively (*P* = 0.001). In conclusion, sarcopenia was not associated with OS in MM patients. High SAT radiodensity and low muscle radiodensity were associated with advanced disease stage and adverse laboratory characteristics.

## Introduction

Multiple myeloma (MM) is a disease that is characterized by wide variability in outcomes, reflecting heterogeneity in both disease-related and host-specific characteristics [1–3]. While disease-specific prognostic factors including cytogenetics are well-established, less is known about the prognostic impact of patient-related characteristics such as body composition. Measures of skeletal muscle and adipose tissue content have been evaluated for their impact on clinical outcomes in several solid and hematologic malignancies through secondary analysis of a single cross-sectional computed tomography (CT) image, most commonly at the third lumbar vertebral level (L3), which provides an estimate of whole body fat and muscle volumes [4]. Sarcopenia, a disorder characterized by a progressive decline in skeletal muscle mass and function, has been shown to occur at a higher frequency in adult cancer patients, especially those with advanced diseases [5]. Radiologic sarcopenia, defined by low skeletal muscle index (SMI) on CT images available from diagnosis or at restaging, has been linked to adverse health outcomes in patients with various malignancies [6–8]. Among hematologic malignancies, sarcopenia has been mostly evaluated in patients with lymphoma, and to a lesser extent in patients with leukemia. While some studies have found sarcopenia to be associated with inferior outcomes with chemoimmunotherapy [8–13] and transplant [14–17], others have shown no prognostic impact [18], or even a protective role of sarcopenia [19]. These discordant results are, at least in part, explained by variability in the populations and subgroups studied and differences in the thresholds used to define sarcopenia [7]. Similarly, CT-derived measures of fat content and distribution have been evaluated for their impact on health outcomes in adult cancer patients [20–22] using various cutoffs and definitions and have reported discordant results. [21, 23–25] The prevalence and impact of muscle and fat content and quality has not been well-established in MM owing to the paucity of available studies, small sample sizes, and heterogeneity in the populations and cutoffs used. ^18^F-fluorodeoxyglucose positron emission tomography/CT (^18^F-FDG PET/CT) obtained at the time of MM diagnosis to assess for lytic lesions provides an opportunity for body composition analysis providing both quantitative and qualitative information on muscle and fat composition. Thus, we designed this study to evaluate the prognostic impact of muscle and fat content and quality assessed by ^18^F-FDG PET/CT in newly diagnosed patients with MM.

## Patients and methods

We included 341 patients diagnosed with MM from January 6th, 2010 to June 20th, 2019, who had a PET/CT performed at our institution within 3 months prior to diagnosis, or after diagnosis but within <1 month of starting treatment. We used a single-slice cross-sectional CT image at the level of L3 for body composition analysis. Segmentation into the muscle and adipose tissue compartments was performed using the semi-automated BodyCompSlicer software, and manual correction was performed, when necessary, by the reviewing radiologist using computer–mouse interface [26]. We used the following CT attenuation thresholds for segmentation: −190 to −30 Hounsfield units (HU) for subcutaneous adipose tissue (SAT), visceral adipose tissue (VAT), and intermuscular adipose tissue (IMAT), and −30 to 150 HU for skeletal muscle tissue. The cross-sectional areas for SAT, VAT, IMAT were calculated in cm^2^. The total adipose tissue (TAT) area was calculated as the sum of the SAT, VAT, and IMAT areas. The subcutaneous fat water area was also measured in cm^2^. The subcutaneous adipose tissue index (SAI), visceral adipose tissue index (VAI), intermuscular adipose tissue index (IMAI), and total adipose tissue index (TAI) were calculated by dividing the cross-sectional area of the respective compartment by height in meters squared (cm^2^/m^2^). The skeletal muscle area (SMA) was calculated as the sum of the paraspinal, abdominal wall, and psoas muscle cross sectional areas (cm^2^). The skeletal muscle index (SMI) was then calculated by dividing the SMA by height in meters squared (cm^2^/m^2^). Muscle and adipose tissue radiodensities were calculated as the mean attenuation in Hounsfield units (HU) of the respective compartments. Patients were classified into 4 categories using body mass index (BMI) in kg/m^2^: underweight (BMI < 18.5), normal weight (BMI: 18.5 to < 25), overweight (BMI: 25 to <30), and obese (BMI ≥ 30).

### Statistical analysis

We used sex-specific medians in our population to divide patients into two groups with low and high SAT, VAT, IMAT, TAT, SAI, VAI, IMAI, TAI, and muscle and fat radiodensities. We also divided patients into three groups using sex-specific tertiles for each of those parameters. Sarcopenia was defined using both sex-specific medians for SMI in our population and using preestablished cutoffs from the literature: <52.4 cm^2^/m^2^ in men and <38.5 cm^2^/m^2^ in women [27], <55 cm^2^/m^2^ in men and <39 cm^2^/m^2^ in women [28], and ≤43.0 cm^2^/m^2^ if BMI is <25 kg/m^2^ or ≤53 cm^2^/m^2^ if BMI is ≥25 in men and <41 cm^2^/m^2^ in women irrespective of BMI [29]. Nominal variables were compared using Fisher’s exact test and continuous variables were compared using the Wilcoxon signed rank test. Univariate and multivariate analysis was performed using Cox proportional hazard models. Variables that were significant on univariate analysis with a two-sided *p*-value of <0.05 were included in the multivariate model. Progression-free survival (PFS) was defined as the time from the start of first-line treatment to first disease progression or death from any cause, whichever occurred first. Patients without an event at the end of follow up were censored. Overall survival (OS) was measured from diagnosis until death from any cause. Patients who were alive at their last follow up were censored. PFS and OS were estimated using the Kaplan–Meier method and groups were compared using the log-rank test. For all statistical analysis, two-sided *p*-values < 0.05 were considered statistically significant. Statistical analysis was performed using JMP® statistical software, Version 16, SAS Institute Inc., Cary, NC, 1989–2022.

## Results

### Baseline characteristics

We included 341 patients. The median age was 65 years; 66% were male. Among all patients, 23%, 65% and 13% had R-ISS stage I, II, and III, respectively. The median BMI was 28.1 kg/m^2^; 37% and 36% were overweight and obese, respectively. Ninety eight percent of included patients received induction with novel agent based regimens, and 54% underwent transplantation. Baseline characteristics are shown in Table 1. The sex-specific medians for muscle and fat parameters at the time of diagnosis are shown in Table 2. The median follow up was 5.7 (95%CI: 5.4–6.3) years. The median PFS in the entire cohort was 37.9 (95%CI: 31.0–55.0) months, and median OS was 7.7 (95%CI: 6.1-Not reached [NR]) years.

**Table 1:** Baseline characteristics.

	Median (IQR)	*N* (%)
**Age (years)**	65 (59–72)	
Age > 65 years		176 (52)
Age > 70 years		108 (32)
Sex (Male)		226 (66)
BMI (kg/m^2^)	28.1 (24.6–31.7)	
Underweight/Normal/Overweight/Obese (*N* = 338)		2 (1)/90 (27)/126 (37)/120 (36)
ISS III (vs. I/II) (*N* = 312)		86 (28)
R-ISS III (vs. I/II) (*N* = 280)		36 (13)
Laboratory data:		
Hb < 10 g/dL (*N* = 306)		78 (25)
Platelets < 150 × 10^9^/L (*N* = 276)		47 (17)
Calcium > 11 mg/dL (*N* = 300)		16 (5)
Creatinine > 2 mg/dL (N = 293)		25 (9)
High LDH IU/L^a^ (*N* = 287)		37 (13)
BMPCs (%) (*N* = 310)	40 (20–69)	
Heavy chain isotype (*N* = 293)		
IgG		170 (58)
IgA		70 (24)
IgM		2 (1)
IgD		3 (1)
None		48 (16)
Light chain isotype (K/L) (*N* = 289)		200 (69)/89 (31)
ASCT		184 (54)
First-line induction (*N* = 323)		
PI		109 (34)
IMiD		85 (26)
PI+IMiD		123 (38)
Other		6 (2)
FISH abnormalities		
HR IgH translocation (*N* = 331)		50 (15)
Trisomy (any) (*N* = 329)		182 (55)
1q gain (*N* = 304)		86 (28)
MYC abnormality (*N* = 301)		27 (9)
Monosomy 13 (*N* = 335)		137 (41)
Del17p/monosomy 17 (*N* = 332)		48 (14)

^a^LDH above the laboratory reference range where the test was performed. > 222 IU/L at Mayo Clinic laboratories. Abbreviations: *ASCT* autologous stem cell transplantation, *BMI* body mass index, *BMPCs* bone marrow plasma cells, *FISH* fluorescence in situ hybridization, *IMiD* immunomodulatory drug, *IQR* interquartile range, *ISS* international staging system, *K* kappa light chain, *L* lambda light chain, *LDH* lactate dehydrogenase, *PI* proteasome inhibitor, *R-ISS* revised international staging system.

**Table 2:** Association between muscle and fat areas and indices and OS in patients with newly diagnosed multiple myeloma.

	Median		OS based on median: above vs. below median - Median (95%CI) (years)	OS based on tertiles: lower vs. middle vs upper - Median (95%CI) (years)
	Males	Females		
SMI (cm^2^/m^2^)	51.8	41.5	7.6 (5.8-NR) vs 9.3 (6.1-NR) *P* = 0.77	6.5 (5.4-NR) vs NR (5.8-NR) vs 7.6 (5.0-NR) *P* = 0.46
IMAT (cm^2^)	30.9	31.1	6.5 (5.4-NR) vs 8.4 (6.1-NR) *P* = 0.27	7.7 (6.1-NR) vs 8.4 (5.4-NR) vs 7.3 (4.6-NR) *P* = 0.56
IMAI (cm^2^/m^2^)	10.2	11.9	6.5 (5.0-NR) vs 9.0 (6.2-NR) *P* = 0.22	9.0 (7.1-NR) vs 5.8 (4.8-NR) vs 7.3 (4.8-NR) *P* = 0.23
SAT (cm^2^)	171.2	227.2	8.4 (6.0-NR) vs 7.6 (5.6-NR) *P* = 0.98	6.1 (4.8-NR) vs NR (7.1-NR) vs 6.5 (4.8-NR) ***P*** = 0.04
SAI (cm^2^/m^2^)	54.6	89.1	7.3 (5.8-NR) vs 7.7 (6.0-NR) *P* = 0.67	7.6 (5.4-NR) vs 9.0 (6.2-NR) vs 7.3 (4.6-NR) *P* = 0.31
VAT (cm^2^)	214.8	122.0	9.0 (6.5-NR) vs 6.5 (5.4-NR) *P* = 0.46	7.6 (5.4-NR) vs 6.3 (4.8-NR) vs NR (9.0-NR) *P* = 0.18
VAI (cm^2^/m^2^)	67.7	48.7	9.0 (6.0-NR) vs 6.5 (5.6-NR) *P* = 0.89	7.3 (5.4-NR) vs 6.5 (5.0-NR) vs NR (6.0-NR) *P* = 0.36
TAT^a^(cm^2^)	428.9	398.9	8.4 (6.0-NR) vs 7.6 (5.8-NR) *P* = 0.85	6.5 (5.1-NR) vs 9.0 (6.0-NR) vs 8.4 (5.8-NR) *P* = 0.61
TAI (cm^2^/m^2^)	139.1	158.3	8.4 (5.8-NR) vs 7.6 (5.8-NR) *P* = 0.82	6.5 (5.1-NR) vs 9.0 (6.0-NR) vs 8.4 (5.8-NR) *P* = 0.50
Muscle radiodensity (HU)	33.5	28.7	8.9 (6.2-NR) vs 7.3 (4.8-NR) *P* = 0.11	5.6 (3.9-NR) vs 7.7 (5.8-NR) vs 9.0 (6.2-NR) *P* = 0.055
SAT radiodensity (HU)	−99.5	−101.9	6.0 (5.1–9.3) vs NR (7.1-NR) ***P*** = 0.003	NR (6.3-NR) vs 9.0 (6.0-NR) vs 5.4 (3.7–8.4) ***P*** = 0.004
SAT water area (cm^2^)	13.7	7.7	6.5 (5.8-NR) vs 9.0 (5.8-NR) *P* = 0.14	NR (5.4-NR) vs 9.0 (5.6-NR) vs 7.1 (4.5-NR) *P* = 0.34

^a^TAT = SAT + VAT + IMAT. Abbreviations: *CI* confidence interval, *IMAT* intramuscular adipose tissue, *IMAI* intramuscular adipose tissue index, *NR* not reached, *OS* overall survival, *SAT* subcutaneous adipose tissue, *SAI* subcutaneous adipose tissue index, *SMI* skeletal muscle index, *TAT* total adipose tissue, *TAI* total adipose tissue index, *VAT* visceral adipose tissue, *VAI* visceral adipose tissue index. *P* values <0.05 are bolded.

### Impact of skeletal muscle content

The prevalence of sarcopenia ranged from 46% to 56% depending on the SMI sex-specific cutoff used [27–29]. Patients with sarcopenia (SMI less than sex-specific median) had lower median BMI (26.1 kg/m^2^) compared to those without sarcopenia (SMI higher than sex-specific median) (BMI = 29.8 kg/m^2^), *P* < 0.001; the prevalence of obesity (BMI > 30) was higher in patients without sarcopenia (48%), compared to those with sarcopenia (24%), *P* < 0.001. Sarcopenia was associated with lower median subcutaneous (165.0 vs. 207.6 cm^2^, *P* < 0.001), visceral (149.8 vs. 191.4 cm^2^, *P* = 0.004), and total (390.4 vs. 445.3 cm^2^, *P* < 0.001) adipose tissue areas. There was no association between sarcopenia and IMAT area (31.2 vs. 30.9 in patients with and without sarcopenia, respectively, *P* = 0.34). Patients with sarcopenia were older (median: 67 vs. 63 years, respectively, *P* < 0.001) and less likely to undergo autologous stem cell transplantation compared to those without sarcopenia (47% vs. 61%, respectively, *P* = 0.02). There was no association between sarcopenia and ISS or R-ISS stage, baseline laboratory characteristics, or cytogenetic characteristics.

Median PFS was 33.4 (95%CI: 27.2–49.8) months in patients with sarcopenia compared to 45.0 (95%CI: 31.9–71.0) months in patients without sarcopenia (*P* = 0.25). There was no difference in PFS between patients with and without sarcopenia among males (31.0 and 45.0 months, respectively, *P* = 0.24), or females (34.5 and 35.3 months, respectively, *P* = 0.80). Similarly, there was no significant difference in PFS between patients with and without sarcopenia among patients <65 years (38.9 and 100 months, respectively, *P* = 0.08) or ≥65 years (33.3 and 31.2 months, respectively, *P* = 0.59). Median OS was 7.6 (95%CI: 5.8-NR) years and 9.3 years (95%CI: 6.1-not reached [NR]) years in patients with and without sarcopenia, respectively (*P* = 0.77) (Table 2). There was no difference in OS between patients with and without sarcopenia among males (6.4 and 5.8, respectively, *P* = 0.72), females (NR in both groups, *P* = 0.99), patients <65 years (9.3 and 9.0 years, respectively, *P* = 0.75) or patients ≥65 years (6.5 and 5.1 years, respectively, *P* = 0.67).

### Impact of adipose tissue content

Among all patients, 1% were underweight, 27% had normal weight, 37% were overweight, and 36% were considered obese. There was no difference in PFS between normal weight (38.9 [95%CI: 29.0–58.9] months), overweight (43.7 [95%CI: 28.6-NR] months), or obese (33.3 [95%CI:27.8–71] months) groups. Similarly, OS was similar between the 3 groups: 6.1 (95%CI: 5.3-NR), 9.0 (95%CI: 6.0-NR), and 7.3 (95%CI: 5.8-NR) years, respectively. There was no association between obesity (BMI > 30 kg/m^2^), SAT, VAT, IMAT, and TAT, and: ISS or R-ISS stage or baseline disease-related laboratory or cytogenetic features.

**Fig. 1:**
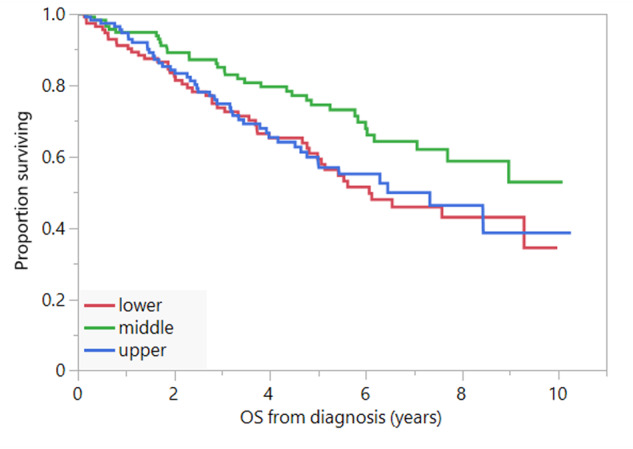
OS and SAT. OS (years) in patients with SAT in the lower tertile (red curve), middle tertile (green curve), and upper tertiles (blue curve)*.* OS overall survival*,* SAT subcutaneous adipose tissue.

Low SAT (below sex-specific median) area was associated with older age compared to high SAT area (above sex-specific median) (median: 66.5 vs. 64 years, *P* = 0.03), while low VAT (median: 63 vs. 66 years, *P* = 0.03), and low IMAT (median: 63 vs. 66 years, *P* = 0.002) areas were associated with younger age. There was no association between high vs. low subcutaneous, visceral, intermuscular, or total adipose tissue areas and indices, and PFS or OS when sex-specific medians were used as cutoffs. Similarly, there was no association between visceral, intramuscular, or total adipose tissue areas and indices, and PFS or OS using sex-specific tertiles. Patients in the middle tertile for SAT area had better OS (NR [95%CI: 7.1-NR]) compared to those in the lower (6.1[95%CI: 4.8-NR years]) and upper (6.5[95%CI: 4.8-NR] years) tertiles (*P* = 0.04) (Fig. 1) (Table 2). There was no difference in PFS between the 3 groups.

Patients with subcutaneous fat water area above the sex-specific median had decreased PFS (31.1 months) compared to those below the median (58.1 months), *P* = 0.04. There was no difference in PFS between patients in the lower (49.8 months), middle (38.9 months) and upper (31.9 months) tertiles for subcutaneous fat water area, *P* = 0.35. Subcutaneous fat water area was not associated with OS when examined as a dichotomous variable (above vs. below the median) or using tertiles.

### Impact of muscle radiodensity

**Fig. 2:**
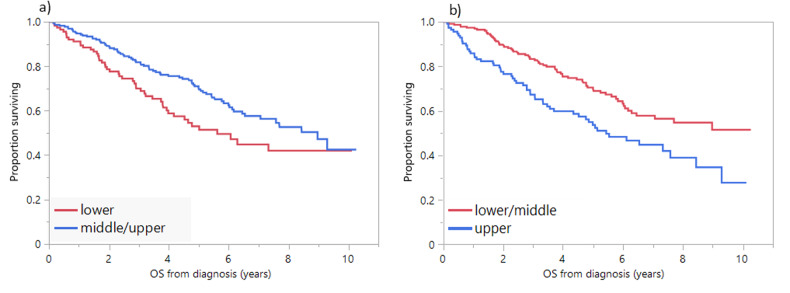
OS and SAT and muscle radiodensity. **a** OS (years) in patients with skeletal muscle radiodensity (HU) in the lower tertile (red curve) compared to those in the middle or upper tertiles (blue curve). **b** OS (years) in patients with SAT radiodensity (HU) in the upper tertile (blue curve) compared to those in the lower or middle tertiles (red curve). HU Hounsfield units*,* OS overall survival*,* SAT subcutaneous adipose tissue.

Low muscle radiodensity was associated with decreased OS; OS was 7.3 years vs. 9.0 years in patients with muscle radiodensity below and above the median, respectively (*P* = 0.11). There was no significant difference in OS between patients in the middle vs. upper tertiles (7.7 vs. 9.0, *P* = 0.52) for muscle radiodensity, but both had significantly increased OS compared to patients in the lower tertile, so these groups (middle and upper tertiles) were combined; median OS was 5.6 years vs. 9.0 years in patients in the lower and middle/upper tertiles for muscle radiodensity, respectively (*P* = 0.02) (Table 2, Fig. 2a). There was no association between muscle radiodensity and PFS. Compared to patients with high muscle radiodensity (middle/upper tertiles), patients with low muscle radiodensity (lower tertile) had higher BMI (median: 30.6 vs. 27.2 mg/m^2^, *P* < 0.001), and higher median subcutaneous (238.6 vs. 168.3 cm^2^, *P* < 0.001), visceral (241.0 vs. 145.2, *P* < 0.001), and intramuscular (50.6 vs. 26.6, *P* < 0.001) adipose tissue areas, but lower SMI (44.2 cm^2^/m^2^ vs. 48.5 cm^2^/m^2^, *P* = 0.01). Patients with low muscle radiodensity were less likely to undergo ASCT (46% vs. 58%, *P* = 0.04). Low muscle radiodensity was associated with higher ISS (III vs. I/II) and R-ISS stages (III vs. I/II), older age (≥65 years), anemia (Hb < 10 g/dL), and renal failure (Cr ≥ 2 mg/dL) (Table 3). On univariate analysis the OS hazard ratio (HR) for the lower tertile of muscle radiodensity was 1.5 (95%CI: 1.1–2.2, *P* = 0.02). On multivariate analysis including age ≥ 65 years, advanced R-ISS, anemia, thrombocytopenia, hypercalcemia, renal failure, and high LDH, the association between low muscle radiodensity and OS did not reach statistical significance (OS HR: 1.5, 95%CI; 1.0–2.3, *P* = 0.07).

**Table 3:** Association between muscle radiodensity and baseline characteristics.

	Low muscle radiodensity (lower tertile) *N* (%)	High muscle radiodensity (middle/upper tertiles) *N* (%)	*P*-value
ISS III (vs. I/II)	41 (39)	45 (22)	**0.002**
R-ISS III (vs. I/II)	19 (20)	17 (9)	**0.023**
Age ≥ 65 years	68 (60)	108 (47)	**0.029**
Hemoglobin < 10 g/dL	40 (40)	38 (19)	**<0.001**
Platelets < 150 × 10^9^/L	14 (16)	33 (18)	0.73
Creatinine ≥ 2 mg/dL	4 (4)	12 (6)	0.59
Calcium ≥ 11 mg/dL	13 (14)	12 (6)	**0.04**
High LDH^a^	12 (13)	25 (13)	1.00

^a^LDH above the laboratory reference range where the test was performed. Greater than 222 IU/L at Mayo Clinic laboratories. Abbreviations: ISS international staging system, LDH lactate dehydrogenase, R-ISS revised international staging system. *P* values <0.05 are bolded.

### Impact of subcutaneous adipose tissue radiodensity

High SAT radiodensity was associated with decreased PFS and OS; PFS was 31.3 months vs. 59.4 months in patients with SAT radiodensity above and below the median, respectively (*P* = 0.005). There was no statistically significant difference in PFS when patients were grouped into lower (55.0 [95%CI: 30.8–75.4] months), middle (33.3 [95%CI: 26.1–71.0] months), and upper (34.0, [95%CI: 28.6–51.5] months) tertiles for SAT radiodensity. OS was 6.0 years vs. NR in patients with SAT radiodensity above and below the median, respectively (*P* = 0.003). There was no difference in OS between patients with SAT radiodensity in the middle vs. lower tertiles (9.0 vs. NR, *P* = 0.86), but both groups had significantly increased OS compared to patients in the upper tertile for SAT radiodensity, so these 2 groups (middle and lower tertiles) were combined. OS was 5.4 years vs. NR in patients with SAT radiodensity in the upper vs. middle/lower tertiles, respectively (*P* = 0.001) (Table 2, Fig. 2b). Compared to patients with lower subcutaneous fat radiodensity (middle/lower tertiles), patients with high radiodensity (upper tertile) had lower BMI (25.3 vs. 29.5, *P* < 0.001), and lower median subcutaneous (140.6 vs. 211.6 cm^2^, *P* < 0.001), visceral (93.6 vs. 216.4 cm^2^, *P* < 0.001), and intramuscular (24.9 vs. 34.0 cm^2^, *P* < 0.001) adipose tissue areas, but higher subcutaneous fat water area (15.3 vs. 9.4 cm^2^, *P* < 0.001). Patients with high SAT radiodensity had higher muscle radiodensity (37.1 HU) compared to those with low subcutaneous fat radiodensity (30.7 HU), *P* < 0.001. There was no difference in SMI between patients with high (47.8 cm^2^/m^2^) and those with low SAT radiodensity (47.1 cm^2^/m^2^), *P* = 0.84.

There was no difference in the rate of ASCT between patients in the upper (51%) vs. middle/lower (55%) tertiles for SAT radiodensity (*p* = 0.56). High SAT radiodensity (upper tertile) was associated with higher ISS (III vs. I/II) and R-ISS stages (III vs. I/II), anemia (Hb < 10 d/dL), thrombocytopenia (platelets < 150 × 10^9^/L), hypercalcemia (calcium ≥ 11 mg/dL), renal failure (Cr ≥ 2 mg/dL), and high LDH. On univariate analysis, the OS HR for the upper tertile of SAT radiodensity was 1.8 (95%CI: 1.3–2.5, *P* = 0.001). On multivariate analysis including age ≥ 65 years, advanced R-ISS, anemia, thrombocytopenia, hypercalcemia, renal failure and high LDH, high SAT radiodensity was independently associated with inferior OS (OS HR: 1.7[95%CI; 1.1–2.6] *P* = 0.03) (Table 4).

**Table 4:** Association between subcutaneous fat radiodensity and baseline characteristics.

	High subcutaneous fat radiodensity (upper tertile) *N* (%)	Low subcutaneous fat radiodensity (middle/lower tertiles) *N* (%)	*P*-value
ISS III (vs I/II)	45 (44)	41 (20)	**<** **0.001**
R-ISS III (vs II)	23 (25)	13 (7)	**<** **0.001**
Age ≥ 65 years	59 (52)	117 (51)	0.91
Hemoglobin < 10 g/dL	37 (36)	41 (20)	**0.005**
Platelets < 150 × 10^9^/L	22 (24)	25 (14)	**0.04**
Creatinine ≥ 2 mg/dL	13 (13)	12 (6)	**0.0495**
Calcium ≥ 11 mg/dL	10 (10)	6 (3)	**0.03**
High LDH^a^	19 (20)	18 (9)	**0.02**

^a^LDH above the laboratory reference range where the test was performed. Greater than 222 IU/L at Mayo Clinic laboratories. Abbreviations: *ISS* international staging system, *LDH* lactate dehydrogenase, *R-ISS* revised international staging system. *P* values <0.05 are bolded.

## Discussion

CT images obtained at diagnosis have been used to provide quantitative and qualitative information on muscle and fat composition in various malignancies. In this study, we evaluated these parameters among patients with newly diagnosed MM. Sarcopenia, defined as skeletal muscle index below the sex-specific median in our population, was associated with lower BMI and lower subcutaneous, visceral, and total adipose tissue, which may reflect a state of cancer-associated cachexia. Patients with low SMI were less likely to undergo ASCT, which may be related to older age of sarcopenic patients. We did not observean association between sarcopenia and PFS or OS even when the analysis was stratified by age and sex. Previous studies have reported discordant results on the prognostic impact of sarcopenia in MM. [23, 30–33] In a retrospective study including 142 patients with MM, sarcopenia (defined as ≤80% high-density muscle within the psoas at the level of L3) was associated with early post-transplant cardiovascular complications, but there was no association with OS [34]. Two other studies showed no association between CT-derived muscle mass and OS [23, 31]. The HOVON 123 study evaluated the impact of both muscle mass and function among 220 patients with MM age ≥75 years; both low skeletal muscle index and decreased muscle function by self-report were associated with early treatment discontinuation and decreased survival [33]. This discordance can be partially explained by variability in the definition of sarcopenia, the thresholds used, and the populations under study.

In this study, we also evaluated measures of fat content, and observed that patients in the upper and lower tertiles for subcutaneous adipose tissue experienced decreased OS compared to those in the middle tertile, suggesting inferior outcomes for both patients with subcutaneous adipopenia and those with obesity. There was no association between BMI, visceral, intramuscular, or total adipose tissue areas, and adverse disease features or survival. Similar to measures of muscle mass, studies evaluating fat content have shown mixed results in various malignancies [20, 21, 23, 25, 35]. In a retrospective study including 56 patients with newly diagnosed MM, low subcutaneous fat index (lower than the median), assessed by CT or PET/CT, was associated with decreased OS, but not PFS, while there was no association between visceral fat index or sarcopenia, and survival. The study also demonstrated that low subcutaneous fat index was associated with higher SUVmax in MM lesions on PET/CT, suggesting that the MM-related hypermetabolic state may account for the association between low SAI and survival [23]. In a subgroup analysis of the GMMG MM5 trial, body composition was assessed on whole body low dose CT for 108 patients and showed that high abdominal VAT was associated with poor treatment response [24]. In a meta-analysis by Aleixo et al. including patients with hematologic malignancies, visceral and subcutaneous adipopenia were both associated with an increased risk of mortality [22]. However, in a subgroup analysis by cancer type, this association was found only in patients with AML, and not in those with lymphoma or MM [22].

Unlike quantitative measures for muscle and adipose tissue, markers of muscle and fat quality correlated with both adverse baseline disease characteristics and with survival in our study; low muscle radiodensity, reflecting higher muscular lipid content [36, 37], was associated higher BMI and adipose tissue content including higher intramuscular fat. It was also associated with adverse disease characteristics and lower likelihood to undergo transplant. On multivariate analysis, its association with OS approached, but did not reach, statistical significance after adjusting for adverse disease characteristics. Low muscle radiodensity has been found to be associated with aging [38], obesity, insulin resistance, and type 2 diabetes [39] in previous studies. In addition, it has been shown to correlate with decreased muscle strength [40], limitation in mobility [41], decreased muscle function independent of muscle mass [40], frailty and functional impairment, [42, 43] and decreased OS [29]. An analysis of CT images at the level of L3 in 185 older adults with cancer showed that high muscle radiodensity was associated with a decreased risk of functional impairment, while SMI was not [43]. It is now increasingly recognized that muscle function, rather than mass, is the main predictor of adverse outcomes [44], which was reflected in the revised definition of sarcopenia by the European Working Group on Sarcopenia in Older People (EWGSOP2) [45]. Physical activity and weight loss have both been shown to reduce fatty infiltration in skeletal muscle [46], and may have a role in improving muscle function.

High SAT radiodensity was associated with adverse disease features, decreased PFS and independently associated with decreased OS in our study. High SAT radiodensity correlated with lower BMI and adipose tissue content, and higher subcutaneous fat water area. The adverse impact of high fat radiodensity is consistent with results from previous studies; in a retrospective study including 91 patients with newly diagnosed MM, high SAT radiodensity on CT or PET/CT was associated with renal failure and lower albumin levels, but was not associated with other baseline laboratory characteristics, age, performance status, or transplant utilization. Similar to our results, high SAT radiodensity was associated with lower BMI and lower SAT and VAT areas and indices, reflecting lower total adipose tissue content. In addition, higher SAT radiodensity was an independent predictor of decreased event-free and overall survival, while SAT and VAT areas did not correlate with survival. High SAT radiodensity was associated with higher levels of proinflammatory cytokines and increased ^18^F-FDG uptake of adipose tissue on PET, suggesting that high SAT radiodensity could reflect a proinflammatory state. The same study did not find an association between low muscle mass or low muscle radiodensity and OS [35]. An increase in adipose tissue radiodensity has been shown to result from a decrease in adipocyte lipid content or adipocyte atrophy [47], increased edema, and/or a phenotypic switch from white to brown adipose tissue. Browning of subcutaneous white adipose tissue can be induced by systemic inflammation, which leads to increased lipid utilization and increased energy expenditure and thermogenesis. This has been postulated to be an early pathophysiologic step in cancer cachexia and precedes muscle atrophy. Thus, inhibition of inflammation may potentially reverse or halt the progression of cancer cachexia [48].

This study is limited by its retrospective nature and potential for selection bias by including only those patients who had a PET/CT performed at our institution at diagnosis, but provides quantitative and qualitative data for muscle and fat composition using low dose CT in a relatively large cohort of patients treated with contemporary regimens and with long term follow up. Future prospective studies are needed to validate our findings and establish uniform definitions and thresholds for muscle and fat parameters.

## Conclusion

Body composition assessed on routine low dose CT images obtained at diagnosis can provide prognostic information in patients with MM. Measures of muscle and fat quality may be better predictors for disease outcomes compared to quantitative measures of muscle and fat content. Understanding the impact of these parameters on health-related outcomes and the underlying pathophysiology has the potential to guide interventions to prevent declines in muscle and fat quality and improve physical function and prognosis in patients with MM.

## Data Availability

The data generated in this study are available upon request from the corresponding author.
